# Draft Genome Sequence of Amikacin- and Kanamycin-Resistant *Mycobacterium tuberculosis* MT433 without *rrs* and *eis* Mutations

**DOI:** 10.1128/genomeA.01363-15

**Published:** 2015-11-19

**Authors:** Angkanang Sowajassatakul, Olabisi O. Coker, Therdsak Prammananan, Angkana Chaiprasert, Saranya Phunpruch

**Affiliations:** a Department of Biology, Faculty of Science, King Mongkut’s Institute of Technology Ladkrabang, Bangkok, Thailand; b Department of Microbiology, Faculty of Medicine, Siriraj Hospital, Mahidol University, Bangkok, Thailand; c National Center for Genetic Engineering and Biotechnology, Tuberculosis Research Laboratory, National Science and Technology Development Agency, Pathum Thani, Thailand; d Drug-Resistant Tuberculosis Research Fund, Siriraj Foundation, Bangkok, Thailand; e Bioenergy Research Unit, Faculty of Science, King Mongkut’s Institute of Technology Ladkrabang, Bangkok, Thailand

## Abstract

We announce the draft genome sequence of amikacin- and kanamycin-resistant *Mycobacterium tuberculosis* MT433, which has been previously described as the strain carrying an unknown resistance mechanism.

## GENOME ANNOUNCEMENT

Epidemic of multidrug-resistant tuberculosis (MDR-TB) and extensively drug-resistant tuberculosis (XDR-TB) causes many global health problems, especially for treatment and control of tuberculosis. In 2013, the World Health Organization (WHO) reported that there were approximately 9 million incident TB cases and 1.5 million deaths from this disease. Of these, approximately 210,000 people died from MDR-TB (1). Recently, genes associated with amikacin (AMK), kanamycin (KM), and capreomycin (CAP) resistances were characterized in 26 XDR-TB and 3 MDR-TB clinical strains. *M. tuberculosis* MT433 is an MDR-TB strain resistant to amikacin and kanamycin, with an MIC of >64 µg/ml. No mutation was found within the genes known to confer resistance to these antituberculous drugs (2). Whole-genome sequencing might be a useful tool to disclose novel mechanisms associated with amikacin and kanamycin resistance in *M. tuberculosis* MT433.

Genomic DNA of *M. tuberculosis* MT433 was isolated by a cetyl trimethylammonium bromide (CTAB) method and sequenced on an Illumina HiSeq platform. The output yielded an average read length of 100 bp and generated 40,836,734 paired-end reads. The sequence reads were aligned to the *M. tuberculosis* H37Rv reference genome (GenBank accession number NC_000962.3) with Bowtie 2 (version 2.1.0). About 99.05% of the reads aligned to the reference genome. The coverage of the reference genome by the reads was investigated by use of BED Tools (version 17.2.0). The result showed that 99.85% of the reference genome was covered by at least one read.

Genome assembly was performed by CLC Genomics Workbench, resulting in a draft genome of 4,409,112 bp with 285 contigs. The contigs have an average length of 15,470 bp, an *N*_50_ of 77,653, and an average coverage of 595×. The GC content of the *M. tuberculosis* MT433 genome is 65.17%. Genome annotation was performed using the NCBI Prokarytoic Genome Annotation Pipeline (PGAP) (http://www.ncbi.nlm.nih.gov/genome/annotation_prok/). *M. tuberculosis* MT433 contains 4,172 genes, 4,084 coding sequences, 35 pseudogenes, and 53 structural RNAs (45 tRNAs, 3 rRNAs, and 5 noncoding RNAs).

The lineage of *M. tuberculosis* MT433 was determined as lineage 4.4.2 (Euro-American) by the PhyTB program (http://pathogenseq.lshtm.ac.uk/phytblive/index.php) (3). In addition, single-nucleotide polymorphisms (SNPs) were determined by the Genome analysis tool kit (GATK) version 3.2 and annotated by SnpEff. *M. tuberculosis* MT433 contains 833 SNPs comprising 342 nonsynonymous, 218 synonymous, and 273 intergenic SNPs compared with the *M. tuberculosis* H37Rv genome.

Our previous report showed that *M. tuberculosis* MT433 did not contain any point mutations in *rrs*, *tap*, *tlyA*, promoter of *eis*, or *whiB7* genes conferring aminoglycoside resistance. The whole-genome sequence results confirmed the unavailability of point mutations in these genes. It is suggested that some other SNPs found in *M. tuberculosis* MT433 might be involved in the unknown drug resistance mechanism. However, it is possible that differential gene expression levels might be responsible for the drug resistance phenotype. Further investigations are needed to confirm this.

### Nucleotide sequence accession numbers.

This whole-genome shotgun project has been deposited at DDBJ/EMBL/GenBank under the accession number LGAX00000000. The version described in this paper is version LGAX01000000.
